# Supplementary material for: Natural variation in yeast reveals multiple paths for acquiring higher stress resistance
Source: BMC Biol. 2024 Jul 4;22:149. doi: 10.1186/s12915-024-01945-7 (PMC11225312; doi:10.1186/s12915-024-01945-7)
Supplement: Supplementary file 2 — Additional file 2: Supplementary Figures. PDF containing Fig. S1. Representative acquired H2O2 resistance assays a panel of diverse yeast strains. Fig. S2. Acquired H2O2 resistance assays for a panel YPS606 transcription factor deletion mutants. Fig. S3. Representative acquired H2O2 resistance assays for all transcription factor mutants. Fig. S4. Representative acquired H2O2 resistance assays for YPS606 gsh1∆ mutants. [file 12915_2024_1945_MOESM2_ESM.pdf]

Supplementary Figures

Natural variation in yeast reveals multiple paths for acquiring higher stress resistance

Amanda N. Scholes[1,2], Tara N. Stuecker[1], Stephanie E. Hood[1], Cader J. Locke[1], Carson L. Stacy[1,2,3], Qingyang Zhang[3], and Jeffrey A. Lewis[1*]

[1] Department of Biological Sciences, University of Arkansas, Fayetteville, Arkansas, United States of America

[2] Interdisciplinary Graduate Program in Cell and Molecular Biology, University of Arkansas, Fayetteville, Arkansas, United States of America

[3] Department of Mathematical Sciences, University of Arkansas, Fayetteville, Arkansas, United States of America

* Corresponding author:
Jeffrey A. Lewis
Department of Biological Sciences
University of Arkansas
850 W. Dickson St., SCEN 601
Fayetteville, AR 72701
E-mail: lewisja@uark.edu
Tel: +1.479.575.7740

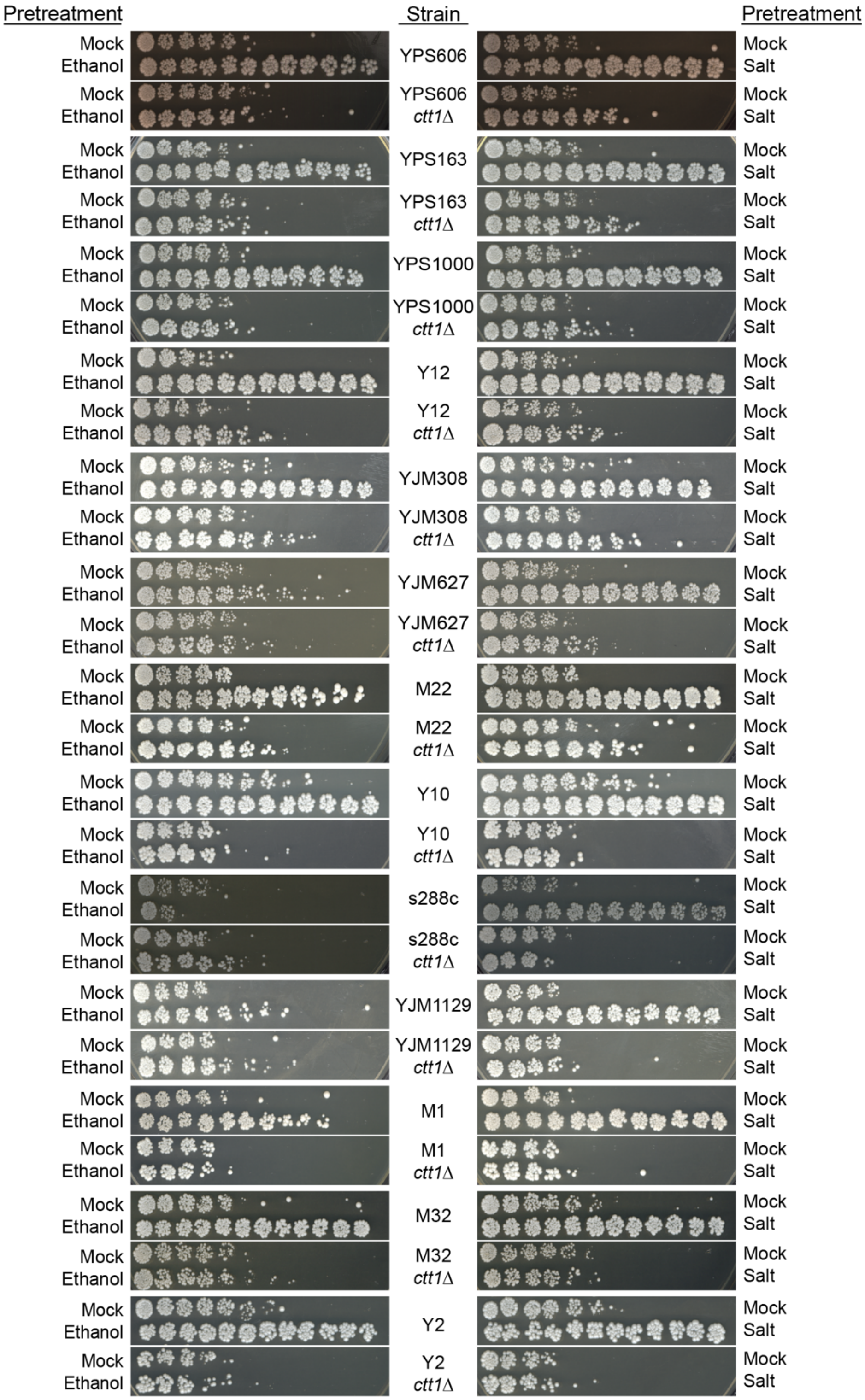

Additional file 2: Fig. S1. Representative acquired H_2O_2 resistance assays a panel of diverse yeast strains. Representative acquired H_2O_2 resistance assays are shown for all strains depicted in Fig. 2.

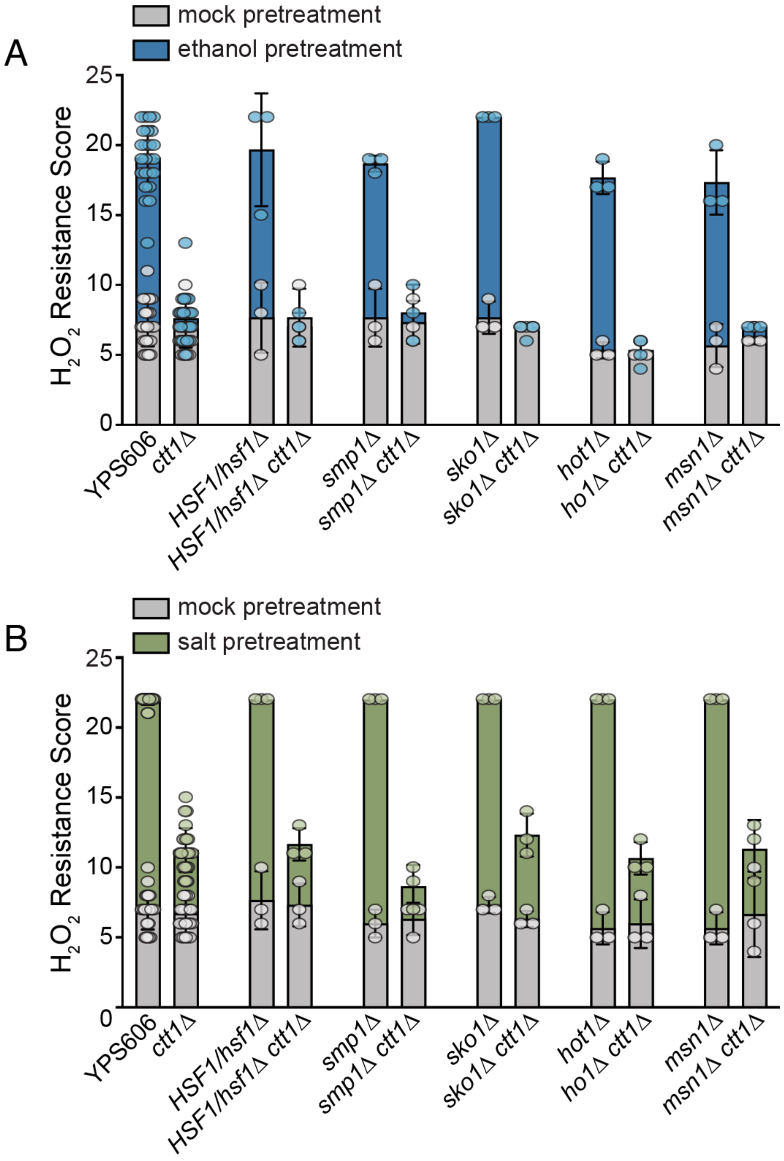

Additional file 2: Fig. S2. Acquired H_2O_2 resistance assays for a panel YPS606 transcription factor deletion mutants. Acquired H_2O_2 resistance assays were performed in homozygous YPS606 *sko1*Δ, *hot1*Δ, and *msn1*Δ mutants plus a heterozygous *HSF1/hsf1*Δ mutant (because *HSF1* is essential). Mutants were assayed in biological triplicate, while wild-type YPS606 and the *ctt1*Δ single mutant were included as controls for each set of experiments and thus had 24 replicates each. Error bars depict the standard deviation (note that the replicates for some strain comparisons had the exact same tolerance score and thus zero standard deviation).

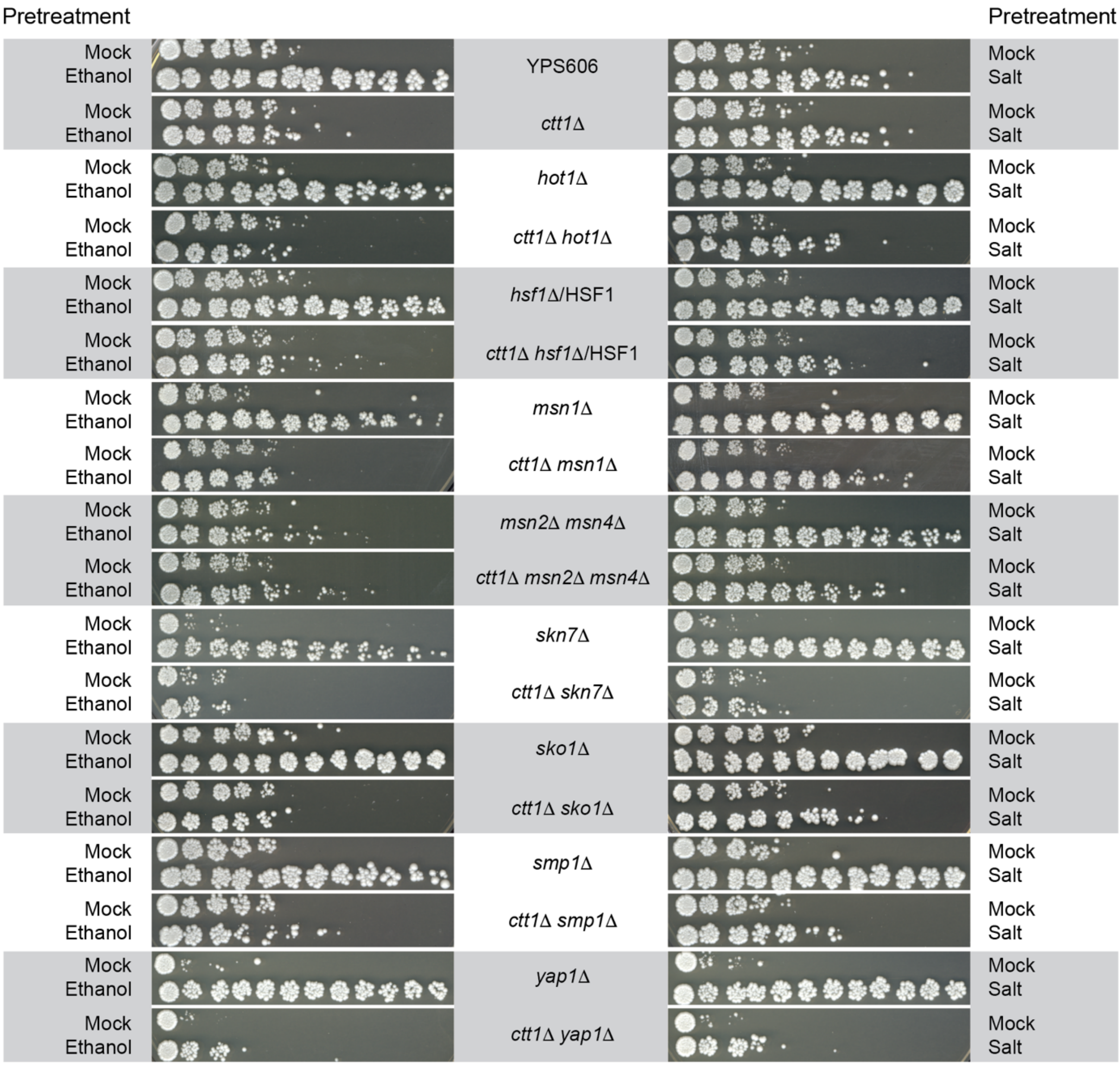

Additional file 2: Fig. S3. Representative acquired H_2O_2 resistance assays for all transcription factor mutants. Representative acquired H_2O_2 resistance assays are shown for all strains depicted in Fig. 6 and Fig. S2.

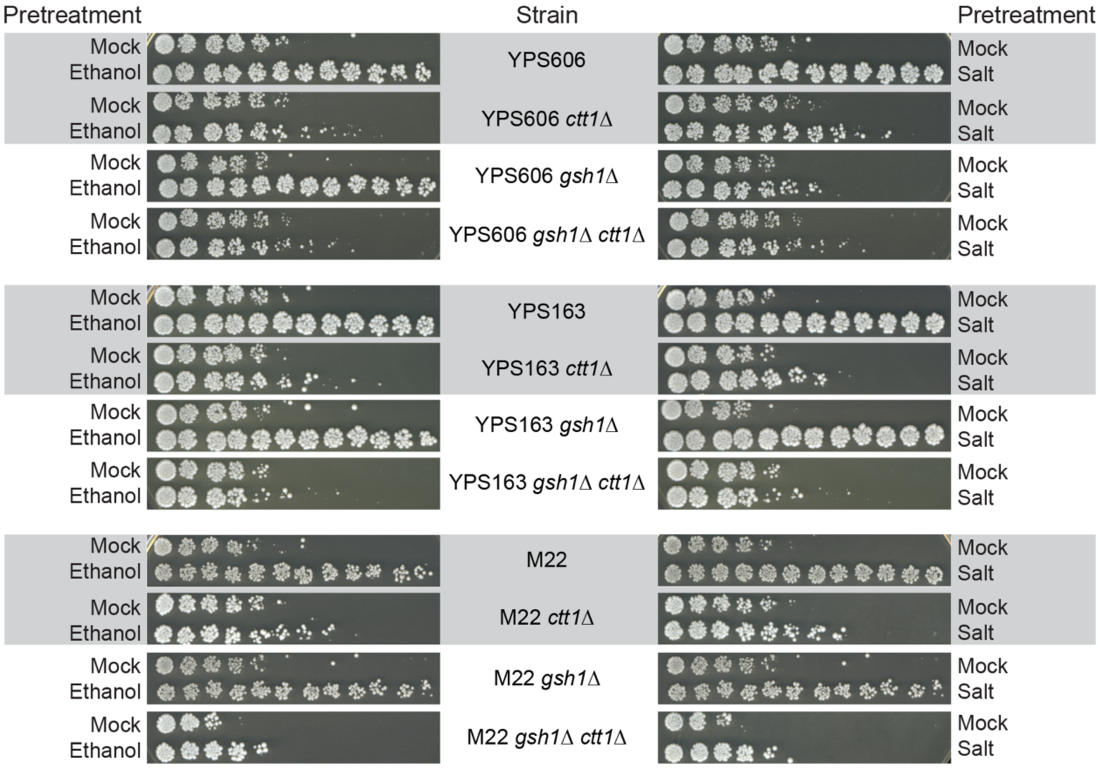

Additional file 2: Fig. S4. Representative acquired H_2O_2 resistance assays for YPS606 *gsh1*Δ mutants. Representative acquired H_2O_2 resistance assays are shown for all strains depicted in Fig. 8.
